# Plasma Cell‐Free DNA Concentration and Fragmentomes Predict Neoadjuvant Chemotherapy Response in Cervical Cancer Patients

**DOI:** 10.1002/advs.202309422

**Published:** 2024-09-25

**Authors:** Ting Peng, Haiqiang Zhang, Lingguo Li, Canhui Cao, Miaochun Xu, Xiaojie Liu, Shitong Lin, Ping Wu, Tian Chu, Binghan Liu, Yashi Xu, Yan Zhang, Yeqin Wang, Jinjin Yu, Wencheng Ding, Xin Jin, Peng Wu

**Affiliations:** ^1^ Department of Obstetrics and Gynecology Union Hospital Tongji Medical College Huazhong University of Science and Technology Wuhan 430000 China; ^2^ National Clinical Research Center for Gynecology and Obstetrics Cancer Biology Research Center (Key Laboratory of the Ministry of Education) Tongji Hospital Tongji Medical College Huazhong University of Science and Technology Wuhan 430000 China; ^3^ BGI Research Shenzhen 518083 China; ^4^ College of Life Sciences University of Chinese Academy of Sciences Beijing 100049 China; ^5^ Department of Obstetrics and Gynecology Tongji Hospital Tongji Medical College Huazhong University of Science and Technology Wuhan 430000 China; ^6^ Department of Obstetrics and Gynecology Affiliated Hospital of Jiangnan University Wuxi Medical College Jiangnan University Wuxi 214000 China

**Keywords:** biomarker, cell‐free DNA, cervical cancer, neoadjuvant chemotherapy

## Abstract

Cervical cancer remains one of the most lethal gynecological malignancies. However, biomarkers for more precise patient care are an unmet need. Herein, the concentration of 285 plasma cell‐free DNA (cfDNA) samples are analyzed from 84 cervical patients and the clinical significance of cfDNA fragmentomic characteristics across the neoadjuvant chemotherapy (NACT) treatment. Patients with poor NACT response exhibit a significantly greater escalation in cfDNA levels following the initial cycle of treatment, in comparison to patients with a favorable response. Distinctive end motif profiles and promoter coverages of cfDNA in initial plasma are observed between patients with differing NACT responses. Notably, the DNASE1L3 analysis further demonstrates the intrinsic association between cfDNA characteristics and chemotherapy resistance. The cfDNA and motif ratios show a good discriminative capacity for predicting non‐responders from responders (area under the curve (AUC) > 0.8). In addition, transcriptional start sites (TSS) coverages around promoters discern the alteration of biological processes associated with chemotherapy resistance and reflect the potential value in predicting chemotherapy response. These findings in predictive biomarkers may optimize treatment selection, minimize unnecessary treatment, and assist in establishing personalized treatment strategies for cervical cancer patients.

## Graphical abstract

Cervical cancer patients with varying responses to neoadjuvant chemotherapy (NACT) exhibit unique patterns of variation in cell‐free DNA (cfDNA) levels and fragmentomic characteristics. The cfDNA emerges as a promising biomarker for predicting tumor response to NACT in cervical cancer, offering a novel approach for monitoring treatment response to optimize clinical management strategies.

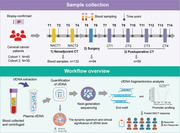

## 1 Introduction

Cervical cancer (CC) remains the most common cancer and a major contributor to mortality for women worldwide, with an annual incidence of ≈604 000 newly diagnosed cases and causing 342 000 deaths in 2020 globally.^[^ ^1^ ^]^ Although the prognosis of localized CC is relatively good, the 5‐year survival rate for advanced, metastatic, or recurrent CC is only 16.5%.^[^ ^2^ ^]^ Additionally, pelvic radiation therapy engenders numerous potential long‐term consequences for cancer survivors, significantly compromising their overall quality of life.^[^ ^3^ ^]^ Hence, it is urgent to explore alternative treatment regimes and biomarkers in order to address this lethal neoplasm and enhance prognostic outcomes effectively.

Since the 1980s, neoadjuvant chemotherapy (NACT) has been widely used to treat various solid tumors. A growing body of evidence from clinical trials supports the superiority of NACT in improving survival outcomes for early and advanced cancer patients.^[^ ^4^, ^5^, ^6^ ^]^ Simultaneously, taxane‐ and platinum‐based chemotherapy in cervical cancer has exhibited a favorable efficacy rate.^[^ ^7^ ^]^ The utilization of NACT offers several potential advantages, including enhancement of local control, elimination of micro‐metastasis,^[^ ^8^ ^]^ and a more favorable toxicity profile compared to concurrent chemoradiotherapy (CCRT).^[^ ^9^, ^10^ ^]^ However, despite its advantages, there are still patients who showed a poor response to this treatment modality, resulting in a delay in the scheduled operation date and severe toxic adverse reactions. Consequently, it is of utmost importance to establish early predictors of treatment response to ensure an effective response in each patient, minimize unnecessary toxicity, and improve patient outcomes.

Recent advances in cell‐free DNA (cfDNA) have opened up an avenue for investigating biomarkers for facilitating the assessment of tumor burden and cancer treatment efficacy.^[^ ^11^ ^]^ For example, Sozzi et al. reported that higher levels of plasma DNA at surgery are indicative of aggressive disease.^[^ ^12^ ^]^ A higher fold increase in cfDNA was found to be an adverse factor for progression in locally advanced hepatocellular carcinoma by Chung et al.^[^ ^13^ ^]^ The cfDNA quantification has been suggested as a valuable tool for indicative of aggressive disease and a higher fold increase in cfDNA level was found to be an adverse factor for progression.^[^ ^12^, ^13^, ^14^ ^]^

The cfDNA fragmentomics is an emerging omics field that encompasses various physical characteristics of cfDNA, such as fragment size, end motifs, and transcriptional start sites (TSS) coverage. It holds great potential for multi‐omics applications, particularly in the context of intracellular gene expression regulation and epigenetics.^[^ ^15^ ^]^ In comparison to circulating tumor DNA (ctDNA), it can provide more comprehensive information about the development and progression of complex diseases through a more sensitive and cost‐effective method.^[^ ^16^, ^17^, ^18^, ^19^, ^20^, ^21^, ^22^ ^]^ An increasing body of research has revealed significant advantages and promising applications of cfDNA fragmentomics in cancer diagnosis and guiding treatment.^[^ ^16^, ^18^, ^23^, ^24^ ^]^

**Figure 1:**
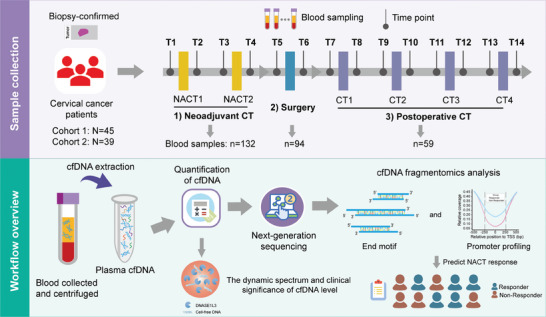
Workflow of this study. This study involved a total of 84 patients who were diagnosed with confirmed cervical cancer through pathological examination. The study population was divided into two cohorts to mutually validate our findings. For cohort 1 of 45 patients, 179 plasma samples were collected before and after treatment. All plasma samples were subjected to cfDNA quantification, and 70 samples underwent whole exome sequencing to analyze cfDNA characteristics. For cohort 2, 106 plasma samples from 39 patients of cohort 2 were collected and subjected to quantification and whole genome sequencing of cfDNA fragmentomes. Overall, these samples were grouped into three distinct “time windows”: 1) neoadjuvant chemotherapy (*n* = 132), 2) surgery (*n* = 94), and 3) postoperative chemotherapy (*n* = 59). Most plasma samples (226 samples, 79.3%) were obtained during the neoadjuvant therapy and surgery stages. This study analyzed the concentration and fragmentomic characteristics of cfDNA in cervical cancer patients before and after treatment and investigated the clinical significance of cfDNA characteristics in early predicting NACT response. NACT: neoadjuvant chemotherapy; CT: chemotherapy.

This study aims to explore the clinical significance of cfDNA concentration and fragmentomes in cervical cancer. Given that this mixture of cfDNA changes across different physiological and pathological states, we first conducted a comprehensive analysis of fluctuations in cfDNA levels in 285 plasma samples from 84 patients with cervical cancer undergoing NACT or not. Our research supplements the understanding of the impact patterns of two major cancer treatment methods (surgery and chemotherapy) on cfDNA levels. Subsequently, we expanded the utilization of cfDNA concentration and fragmentomes including end motifs and TSS coverage as early predictive tools for assessing the response to NACT in patients with cervical cancer (**Figure** **1**). Lastly, we discussed the crucial role of DNASE1L3 in shaping cfDNA characteristics and chemotherapy resistance.

## 2 Results

### 2.1 Characteristics of Patients and Samples

A total of 285 plasma samples from 84 patients with histologically confirmed diagnoses of cervical cancer were included in this study. We divided the study population into two cohorts to mutually validate our findings (See Experimental Section, Figure 1). The time points of blood sample collection are detailed in Figure S1a,b (Supporting Information). Overall, the samples were grouped into three distinct “time windows”: 1) neoadjuvant chemotherapy, 2) surgery, and 3) postoperative chemotherapy. The majority of plasma samples (226 samples, 79.3%) were obtained during the neoadjuvant therapy and surgery stages. **Tables** **1** and **2** presented the individual characteristics of patients in cohort 1 and cohort 2, respectively. In cohort 1, the main histological type of cervical cancer patients was squamous cell carcinoma (82.2%). The proportions of cervical cancer patients in stages I, II, and III are 31.1%, 53.3%, and 15.6%, respectively. In cohort 2, squamous cell carcinoma remains the most common histological subtype (87.2%). The distribution of cervical cancer patients across stages I, II, and III is 33.3%, 25.7%, and 41.0%, respectively. Out of the 84 patients, 46 patients (54.8%) with stage IB3–III underwent platinum‐based neoadjuvant chemotherapy. The clinical response criteria according to the Response Evaluation Criteria in Solid Tumours (RECIST) were used to determine the responsiveness to neoadjuvant chemotherapy (See Experimental Section). Figure S1c (Supporting Information) illustrates the number of samples that undergo the quantification of plasma cfDNA level and the following sequencing in the two cohorts.

**Table 1:** The clinicopathological features of patients in cohort 1.

Clinical characteristics		Patients [*n*]	Percentage [%]
Age (years)			
	≤50	21	46.7
	>50	24	53.3
Histology			
	SCC	37	82.2
	AC	5	11.1
	NEC	3	6.7
Stage (FIGO 2018)			
	I	14	31.1
	II	24	53.3
	III	7	15.6
Tumor size (cm)			
	≤4	25	55.6
	>4	20	44.4
Grade			
	Grade 1	2	4.4
	Grade 2	17	37.8
	Grade 3	19	42.2
	NC	7	15.6
Treatment			
	NACT	22	48.9
	Non‐NACT	23	51.1
NACT response			
	Responder	12	26.7
	Non‐Responder	6	13.3
	Unable to classify	4	8.9

NC: Not clear; NACT: Neoadjuvant chemotherapy; FIGO: International Federation of Gynecology and Obstetrics; SCC: Squamous cell carcinoma; AC: Adenocarcinoma; NEC: Neuroendocrine carcinoma.

**Table 2:** The clinicopathological features of patients in cohort 2.

Clinical characteristics		Patients [*n*]	Percentage [%]
Age (years)			
	≤50	15	38.5
	>50	24	61.5
Histology			
	SCC	34	87.2
	AC	4	10.2
	ASC	1	2.6
Stage (FIGO 2018)			
	I	13	33.3
	II	10	25.7
	III	16	41.0
Tumor size (cm)			
	≤4	23	59.0
	>4	16	41.0
Grade			
	Grade 2	12	30.8
	Grade 3	16	41.0
	NC	11	28.2
Treatment			
	NACT	24	61.5
	Non‐NACT	15	38.5
NACT response			
	Responder	12	30.8
	Non‐Responder	9	23.1
	Unable to classify	3	7.7

NC: Not clear; NACT: Neoadjuvant chemotherapy; FIGO: International Federation of Gynecology and Obstetrics; SCC: Squamous cell carcinoma; AC: Adenocarcinoma; ASC: Adenosquamous carcinoma.

### 2.2 The Dynamic Spectrum of cfDNA Levels at Various Stages of Surgery and Chemotherapy in CC Patients

**Figure 2:**
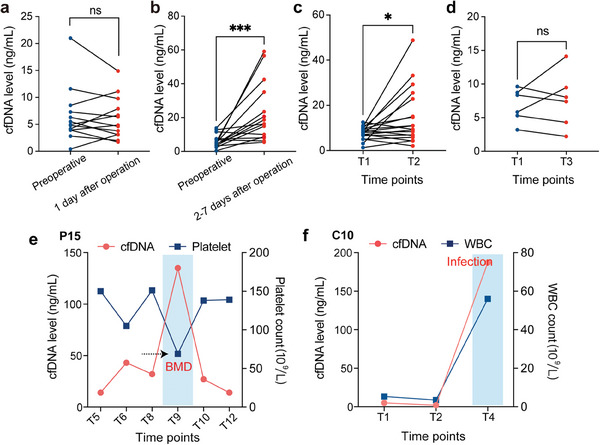
The cfDNA level during surgery and chemotherapy in cohort 1. a) Comparison of cfDNA levels one day after surgery to preoperative levels (*n* = 13). b) Comparison of cfDNA levels 2 to 7 days after surgery to preoperative levels (*n* = 15). c) Comparison of cfDNA levels (*n* = 18) between one day after the first cycle of NACT (T2 time point) and pretreatment (T1 time point). d) Comparison of cfDNA levels (*n* = 6) between three weeks after the first cycle of chemotherapy (before the second cycle of NACT, T3 time point) and pretreatment (T1 time point). e) Patient P15: cfDNA levels during chemotherapy, exhibiting a significant surge at BMD. f) Patient C10: cfDNA levels during chemotherapy, revealing a sharp increase during severe infection. The *p* values were determined by the Wilcoxon test: ns, not significant; *p < 0.05; **p < 0.01; ***p < 0.001; ****p < 0.0001.

As cfDNA was mainly released into circulation by cell death, its level may be influenced by several clinicopathological factors.^[^ ^25^ ^]^ To reveal the dynamic spectrum and clinical significance of cfDNA concentration during treatment, we analyzed a total of 285 plasma samples from 84 patients with cervical cancer undergoing NACT or not. The changes in cfDNA levels during surgery and chemotherapy in cohort 1 are shown in **Figure** **2**. The results showed that compared to preoperative levels, no significant change in cfDNA levels 1 day after the operation (Wilcoxon test, *p* = 0.8394) (Figure 2a) while a significant increase in levels from 2 to 7 days (median: 16.27, range: 5.44–59.07) after the operation (median: 4.45, range: 0.42–13.85) (Wilcoxon test, *p* = 0.0006) (Figure 2b). Conversely, cfDNA levels significantly increased on day 1 after the first cycle of NACT (T2 time point) compared to prechemotherapy (T1 time point) levels (Wilcoxon test, *p* = 0.0237) (Figure 2c) and cfDNA levels returned to baseline at T3 subsequently (the time point before the commencement of the second cycle of NACT) (Figure 2d). The temporal changes of specific cfDNA levels were also observed in cohort 2 (Figure S2a–d, Supporting Information). In addition, when bone marrow depression (BMD) occurred in patient P15 of cohort 1, there was a significant rise in the cfDNA level (Figure 2e, cfDNA level = 134.5 ng mL^−1^; Platelet count < 100 × 10^9^ L^−1^). Similarly, we identified a significant increase in cfDNA levels in the condition of severe infection (Figure 2f, cfDNA level = 187.0 ng mL^−1^; white blood cell (WBC) > 50 × 10^9^ L^−1^). Equally, in cohort 2, the rise in cfDNA levels was observed, regardless of whether patients were experiencing BMD or infection (Figure S2e–h, Supporting Information). These findings revealed several confounding factors that potentially have a significant impact on the results of cfDNA analysis.

### 2.3 cfDNA Concentration Analysis in CC Patients with Different NACT Responses

**Figure 3:**
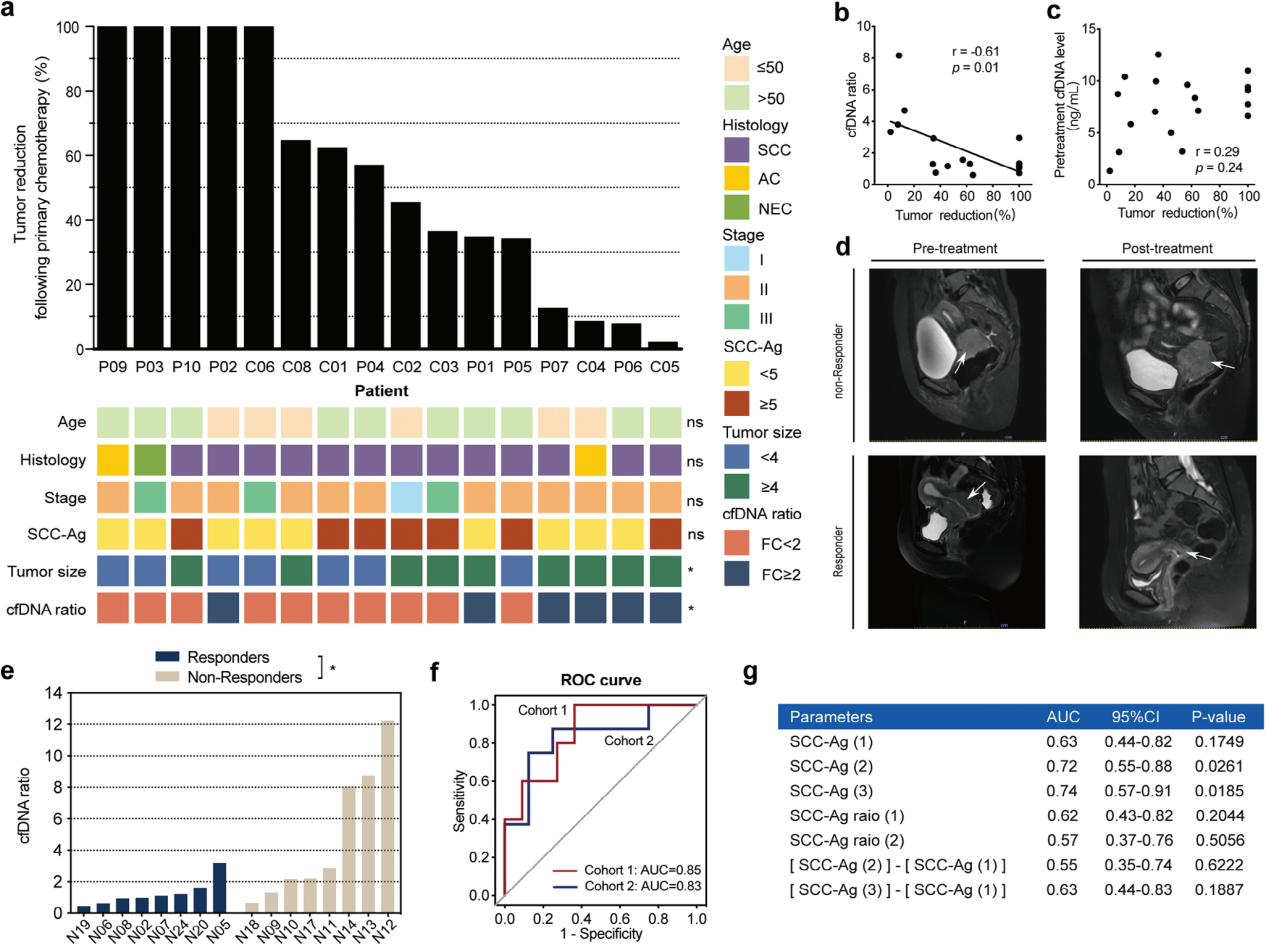
The cfDNA concentration analysis in cervical cancer patients with different neoadjuvant chemotherapy (NACT) responses. a) Association of clinical characteristics and the cfDNA ratio with the percentage of tumor reduction after the first cycle of NACT in patients in cohort 1 (*n* = 16). SCC: Squamous cell carcinoma; AC: Aenocarcinoma; NEC: Neuroendocrine carcinoma. The *p* values were determined by Mann–Whitney U test: ns, not significant; *p < 0.05. b) Correlation between the percentage of tumor reduction after one cycle of chemotherapy and the cfDNA ratio (T2/T1) in cohort 1 (*n* = 16, Spearman r = −0.61). c) Correlation between the percentage of tumor reduction after one cycle of chemotherapy and the initial cfDNA level in cohort 1 (*n* = 18, Spearman r = 0.29). d) Representative radiographic images of patients achieving NACT response and non‐response. e) The cfDNA ratio in non‐responders and responders in cohort 2 (*n* = 16, Mann–Whitney U test). f) Receiver operator characteristic (ROC) curves of early detection performance for non‐responders in cervical cancer patients using cfDNA ratio in cohort 1 and cohort 2. g) AUC and 95% CI for various SCC‐Ag parameters. SCC‐Ag (1) represents SCC‐Ag level before treatment, SCC‐Ag (2) represents SCC‐Ag level after one cycle of NACT, SCC‐Ag (3) represents SCC‐Ag level after two cycles of NACT, SCC‐Ag ratio (1) represents the ratio of SCC‐Ag (2) to SCC‐Ag (1), SCC‐Ag ratio (2) represents the ratio of SCC‐Ag (3) to SCC‐Ag (1), [SCC‐Ag (2)] – [SCC‐Ag (1)] represents SCC‐Ag (2) minus SCC‐Ag (1), [SCC Ag (3)] – [SCC‐Ag (1)] represents SCC‐Ag (3) minus SCC‐Ag (1).

We next explored whether the cfDNA levels were associated with NACT response. In this analysis, we focused on the 22 patients of cohort 1 receiving NACT (12 responders, 6 non‐responders, and 4 unable to classify) and 24 patients who received NACT in cohort 2 (12 responders, 9 non‐responders, and 3 unable to classify). The sample with missing values was excluded. To figure out the relationship between clinical parameters and chemotherapy sensitivity, we first analyzed the association of age (≤ 50, > 50), histology (squamous cell carcinoma, adenocarcinoma, neuroendocrine carcinoma), stage (I, II, III), squamous cell carcinoma antigen (SCC‐Ag ≥ 5 ng mL^−1^, < 5 ng mL^−1^), and tumor size (< 4 cm, ≥ 4 cm) with the percentage of tumor reduction after the first cycle of NACT in the assessable patients from the cohort 1 (*n* = 16). The results showed no statistically significant difference between these clinical characteristics (age, histology, stage, SCC‐Ag level) and tumor reduction besides tumor size. **Figure** **3**a showed that there was a significant difference in tumor reduction among patients with different initial tumor sizes (Mann–Whitney U test, *p* = 0.029). This relationship between initial tumor size and the proportion of tumor shrinkage may be due to the limited number of tumor cells killed by each chemotherapy treatment for a larger tumor. Moreover, we observed that patients with a poor response to NACT showed a notably higher increase in cfDNA levels following the initial cycle of treatment compared to those with a favorable response. We defined the ratio of cfDNA levels on day 1 after the first cycle of NACT (T2 time point) to before treatment (T1 time point) as the cfDNA ratio. A significant discrepancy in tumor reduction was evident between the group with a cfDNA ratio ≤ 2 and the group with a cfDNA ratio > 2 (Mann–Whitney U test, *p* = 0.021) (Figure 3a). There was a significantly negative correlation between the tumor reduction and the cfDNA ratio (Spearman r = −0.61, *p* = 0.01) (Figure 3b). However, the initial level of cfDNA was independently associated with the response to chemotherapy (Spearman r = 0.29, *p* = 0.24) (Figure 3c). To validate the effectiveness of the cfDNA ratio as a reflection of NACT response, we further compared the cfDNA ratios between responders and non‐responders in cohort 2. Figure 3d showed representative radiographic images of patients achieving response and non‐response. In this analysis, three patients were excluded due to insufficient clinical information for response evaluation. Additionally, based on our previous findings on confounding factors, five patients were excluded including one patient with obvious infections, three patients experiencing bone marrow depression after chemotherapy, and one patient with interference from cardiac surgery during NACT. As shown in Figure 3e, the non‐responders (*n* = 8) also showed generally higher cfDNA ratios than the responders (*n* = 8) (Mann–Whitney U test, *p* = 0.028). These findings suggested a positive correlation between elevated cfDNA levels after treatment and poorer NACT response.

To determine the performance of the cfDNA ratio in early predicting non‐responders, we plotted the receiver operating characteristic (ROC) curves based on the probability of non‐responders by the cfDNA ratio (Figure 3f). For cohort 1, the cfDNA ratio showed a good discriminative capacity for the prediction of non‐responders from responders with an AUC of 0.85 (95% CI, 0.66–1.00), a result further confirmed in cohort 2 (AUC = 0.83). Since SCC‐Ag levels as a serologic tumor indicator for cervical cancer in clinical practice, we compared the performance of SCC‐Ag at different time points with the cfDNA ratio in predicting non‐responders. Among these SCC‐Ag parameters, SCC‐Ag (1) represents SCC‐Ag level before treatment, SCC‐Ag (2) represents SCC‐Ag level after the first cycle of NACT, SCC‐Ag (3) represents SCC‐Ag level after the second cycle of NACT, SCC‐Ag ratio (1) represents the ratio of SCC‐Ag (2) to SCC‐Ag (1), SCC‐Ag ratio (2) represents the ratio of SCC‐Ag (3) to SCC‐Ag (1). This group included 24 responders and 15 non‐responders. Figure 3g displays the AUCs for the various SCC‐Ag parameters. The AUCs for these parameters ranged from 0.55 to 0.74. SCC‐Ag (3), which represents the SCC‐Ag level after two cycles of NACT, exhibited the best discriminative performance (AUC = 0.74, 95% CI: 0.57–0.91). In contrast, the cfDNA ratio displayed superior performance to the SCC‐Ag on early identification of non‐responders. These results demonstrate that the cfDNA ratio is closely related to patient responsiveness to chemotherapy, and it can serve as a timely assessment indicator for response to NACT in cervical cancer.

Furthermore, it was observed that among the five patients diagnosed with squamous cervical cancer who exhibited poor response to NACT within cohort 1, three non‐responder cases (P07, C05, P05) consistently displayed increased levels of plasma cfDNA even after two cycles of NACT (P07: T4/T1 = 6.20; C05: T4/T1 = 7.85; P05: T4/T1 = 2.83). Conversely, SCC‐Ag levels tended to exhibit an abnormal decrease (Figure S3a–c, Supporting Information). Similarly, within cohort 2, two non‐responder patients with squamous cervical cancer (N13, N17) demonstrated significant decreases in SCC‐Ag levels, while both exhibited elevated cfDNA levels following treatment (NA13: T4/T1 = 4.43; NA17: T4/T1 = 3.15) (Figure S3d,e, Supporting Information). These findings revealed the dysregulated cfDNA digestion in non‐responders and the potential clinical usage of cfDNA concentration for predicting the tumor response to NACT in CC patients.

### 2.4 Plasma cfDNA End Motifs in CC Patients with Different NACT Responses

**Figure 4:**
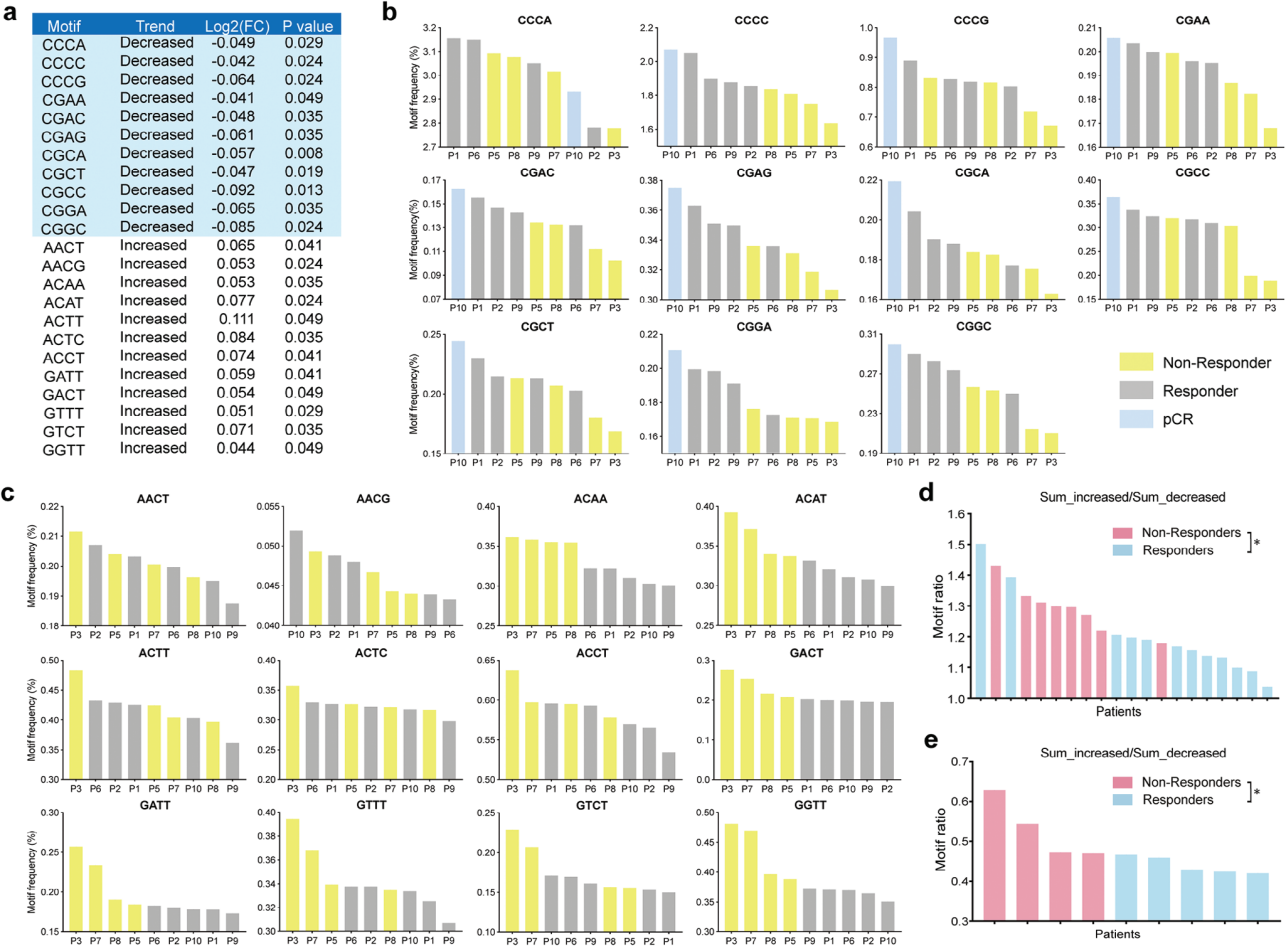
Plasma cfDNA end motifs in cervical cancer patients with different NACT responses. a) Differential cfDNA 5′end motifs (11 down‐regulated end motifs and 12 up‐regulated end motifs) between 12 responder patients and 8 non‐responder patients of cohort 2. b) Distribution of frequency of 11 down‐regulated end motifs in nine patients receiving NACT of cohort 1. c) Distribution of frequency of 12 up‐regulated end motifs in nine patients receiving NACT of cohort 1. d) The motifs ratio in 12 responders and eight non‐responders of cohort 2. e) The motifs ratio in five responders and four non‐responders from cohort 1. The motif ratio was calculated between the accumulated frequencies of significantly increased motifs and decreased motifs. The *p* values were determined by the Mann–Whitney U test: ns, not significant; *p < 0.05; **p < 0.01; ***p < 0.001; ****p < 0.0001.

The nucleotide sequences at the end of cfDNA fragments, known as end motifs, have been reported to reflect cfDNA cleavage preference and nuclease activity.^[^ ^18^, ^25^ ^]^ We hypothesized that the dysregulated cfDNA digestion observed in non‐responders could be manifested not only in the concentration of cfDNA in plasma but also in the end motif of plasma cfDNA. To assess the characteristics of cfDNA fragmentomes in patients responding or not responding to NACT, we performed next‐generation sequencing of serial cfDNA samples (Figure 1c). Since genome‐wide data could offer a more comprehensive reflection of these features, we therefore initially analyzed these features by using whole genome sequencing (WGS) data. We deciphered the distribution of 256 types of 4‐mer ends motif in the plasma cfDNA of 12 responder patients and 8 non‐responder patients in cohort 2 (three patients were excluded due to the lack of sufficient tumor size information, and one patient was excluded due to severe cardiovascular disease) and identified 11 significantly increased (Log_2_(fold change) > 0.04) and 12 decreased motifs (Log_2_(fold change) < −0.04) in non‐responder patients compared with the responder patients (Mann–Whitney U test, *p*‐value < 0.05) (**Figure** **4**a). It was noteworthy that all significantly increased motifs started with adenine (A) and guanine (G) deoxynucleotide, while all significantly decreased motifs started with cytosine (C) deoxynucleotide. To validate this result, we examined the frequency distribution of these identified differential motifs in plasma samples of cohort 1 by whole‐exome sequencing (WES), which included 5 responders and 4 non‐responders (one patient was excluded due to the absence of clinical data for classification). These frequency patterns of the differential motifs exhibited similar trends between non‐responder and responder patients (Figure 4b,c). It was noteworthy that the patient with pathologic complete response (pCR) exhibited the highest frequencies among all responders in most of the top decreased motifs (10 out of 11) (Figure 4b). In contrast, the patient with a poor prognosis pathological type of cervical cancer (P3) has the lowest frequencies in all the top decreased motifs (Figure 4b).

To effectively predict NACT response, we introduced the concept of “motif ratio,” which represents the ratio between the accumulated frequency of significantly increased and decreased motifs specific to non‐responders identified in cohort 2. As shown in Figure 4d, the majority of non‐responder patients exhibited higher motif ratios (median: 1.297, range: 1.178–1.431) in comparison to responder patients (median: 1.162, range: 1.036–1.502) (*p* = 0.0201) in cohort 2. This pattern was further validated in cohort 1 (Figure 4e). These results highlight the strong association between the ends motif and the NACT response in cervical cancer patients and suggest that the motif ratio holds potential in distinguishing different NACT response groups.

### 2.5 Promoter Coverage Profiles of Plasma cfDNA in CC Patients with Different NACT Responses

**Figure 5:**
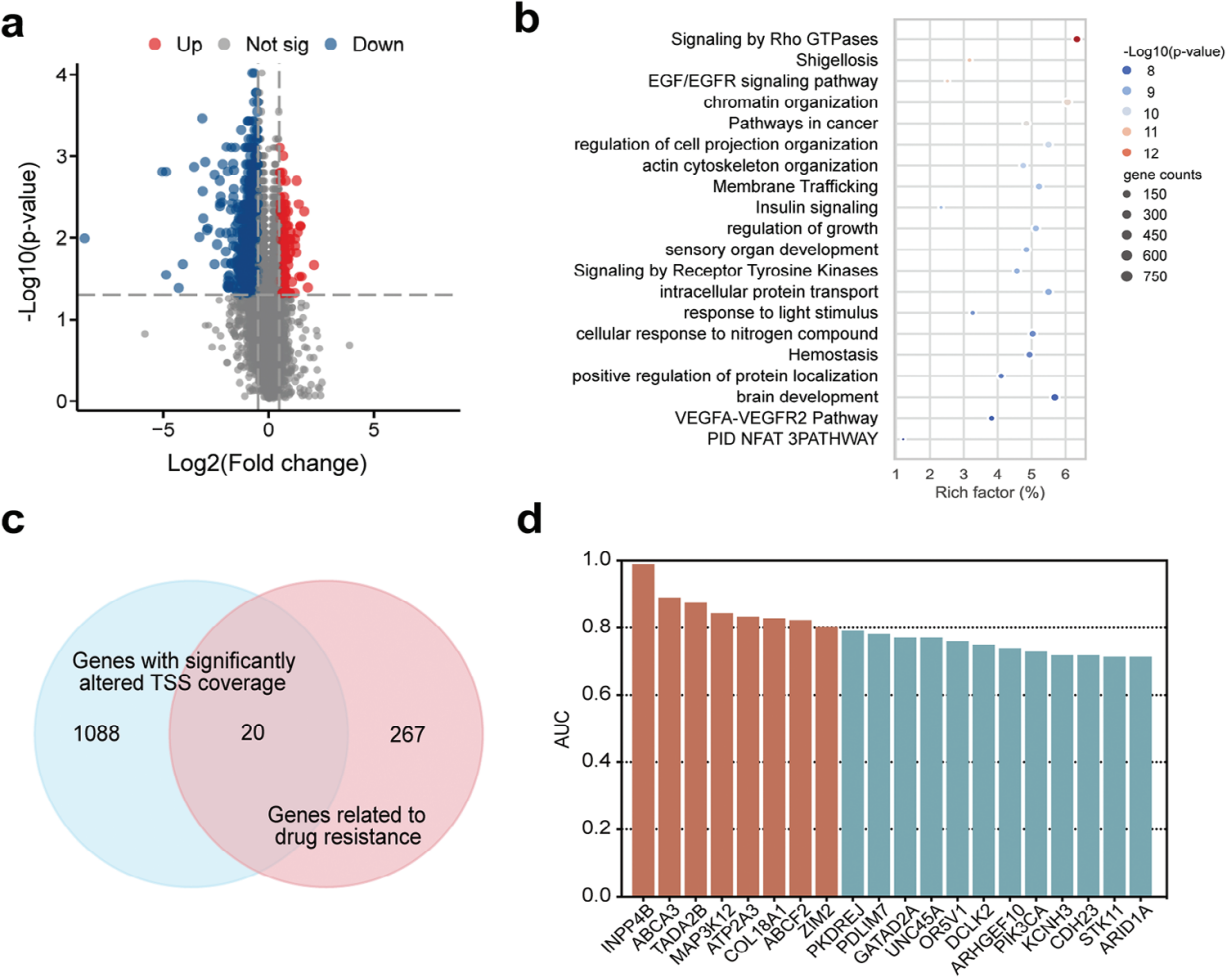
Promoter coverage profiles of plasma cfDNA in cervical cancer patients with different NACT responses. a) A volcano plot showing declined and elevated TSS coverage in non‐responder patients (*n* = 8) compared with responder patients (*n* = 12). The *p* values were determined by the Mann–Whitney U test: Log_2_(fold change) ≥ 0.5, Log_2_(fold change) ≤ − 0.5, *p* < 0.05. b) The top 20 enriched pathways of the dysregulated genes of the non‐responder group in our study. c) A Venn diagram of the intersection between genes with significantly differential coverage of TSS region and drug‐resistant genes related to the occurrence of NACT resistance in cervical cancer. d) Predictive performance of TSS scores of 20 drug‐resistant genes on differentiating non‐response in cervical cancer patients receiving NACT. The box plot shows the corresponding AUC for each gene. AUC: area under the curve; TSS: transcriptional start site.

It has been reported that the cfDNA coverages in the region around TSS were associated with the gene expression level. High TSS coverage indicates a low gene expression level, while low TSS coverage indicates a high gene expression level.^[^ ^21^ ^]^ To explore more possibilities of cfDNA fragments in predicting clinical chemotherapy response, we analyzed the TSS coverage profiles of cfDNA at pretreatment (T1 time point) among 20 patients with different NACT responses in cohort 2 by WGS. To infer the differentially expressed genes between non‐responder patients and responder patients, we compared the TSS coverage of all genes between these two groups through the use of the TSS score (See Experimental Section). Compared with responder patients, 131 and 980 genes showed significantly increased (Log_2_(fold change) ≥ 0.5) and decreased (Log_2_(fold change) ≤ −0.5) TSS scores in non‐responder patients (*p* < 0.05), respectively (**Figure** **5**a). These genes were inferred as the genes with significantly reduced and elevated expression in this non‐responder group. Figure S4a (Supporting Information) shows the unsupervised clustering of the differentially expressed genes among patients with and without NACT response based on TSS scores. The responders and non‐responders tended to gather into two distinct clusters, indicating that the expressions of these genes were highly specific to the NACT response.

Moreover, we utilized Metascape^[^ ^26^ ^]^ (https://metascape.org/) for a comprehensive pathway enrichment analysis on the dysregulated genes of the non‐responder group. The top 20 enriched pathways are shown in Figure 5b. These findings revealed that the dysregulated genes were predominantly enriched in the pathways of biological processes affected by cancer progression and chemotherapy resistance, including signaling by Rho GTPases,^[^ ^27^ ^]^ EGF/EGFR signaling pathway,^[^ ^28^ ^]^ chromatin organization,^[^ ^29^ ^]^ pathways in cancer, insulin signaling,^[^ ^30^ ^]^ regulation of growth, signaling by Receptor Tyrosine Kinases,^[^ ^31^ ^]^ VEGFA‐VEGFR2 signaling (Figure 5b).^[^ ^32^, ^33^ ^]^ These pathways may be associated with low sensitivity of tumor cells to chemotherapy drugs in tumors by regulating mechanisms such as cell cycle, proliferation, and apoptosis, indicating the development of chemotherapy resistance and the associated biological processes in non‐responder patients.

Furthermore, we explored the enriched pathways in the differential genes between the non‐responder and responder groups identified at T2, and T4 time points during NACT. (Figure S4c,d, Supporting Information). In addition to the pathways mentioned above that are involved in mediating chemotherapy resistance, there are many other enriched pathways associated with tumor survival and proliferation. For example, positive regulation of cell development, regulation of cellular catabolic process, cellular responses to stress, and cellular response to chemical stress (Figure S4c,d, Supporting Information). Moreover, several enriched pathways have been evidenced to participate in malignant tumor behavior, therapeutic resistance, and clinical outcomes including ribosome biogenesis in eukaryotes,^[^ ^34^, ^35^ ^]^ MAPK1/MAPK3 signaling,^[^ ^36^ ^]^ transcriptional activation by NRF2 in response to phytochemicals,^[^ ^37^ ^]^ and positive regulation of phosphorylation (Figure S4c,d, Supporting Information).^[^ ^38^ ^]^ These findings indicate the development of chemotherapy resistance and the underlying biological processes in non‐responder patients. Subsequently, we conducted a pathway analysis on the dysregulated genes reported by Xun et al.^[^ ^39^ ^]^ that are associated with chemotherapy resistance in patients undergoing NACT to further validate our results. As shown in Figure S4b (Supporting Information), we revealed that NACT‐resistant patients exhibited dysregulation in multiple interconnected networks, including the RHO GTPase cycle, actin filament‐based processes, protein phosphorylation, regulation of cellular response to stress, chromatin organization, and more. It is worth noting that a significant overlap exists between these networks and the identified pathways of our study (Figure 5b; Figure S4c,d, Supporting Information). These results suggest that the enriched pathways identified through cfDNA coverages around promoters discerned the alteration of biological processes associated with chemotherapy resistance.

In addition, 20 genes were co‐identified in our investigation of NACT response and Xun et al's paper (Figure 5c).^[^ ^39^ ^]^ Among the 20 genes, 18 genes tend to have a higher TSS score (INPP4B, ABCA3, TADA2B, ATP2A3, COL18A1, ABCF2, ZIM2, MAP3K12, GATAD2A, PDLIM7, OR5V1, PKDREJ, ARHGEF10, DCLK2, KCNH3, STK11, ARID1A, CDH23) and 2 genes tend to have a lower TSS score (UNC45A, PIK3CA) in non‐responder group. Based on these 20 co‐identified genes, we obtained high AUCs (median: 0.78, range: 0.71–0.99) in the differentiation between the responder and non‐responder patients. Among these genes, the INPP4B gene showed the highest AUC of 0.99 (Figure 5d). This reflects the potential value of TSS scores in predicting chemotherapy response.

### 2.6 Predictive Performance of Clinical and cfDNA Features on NACT Response

**Figure 6:**
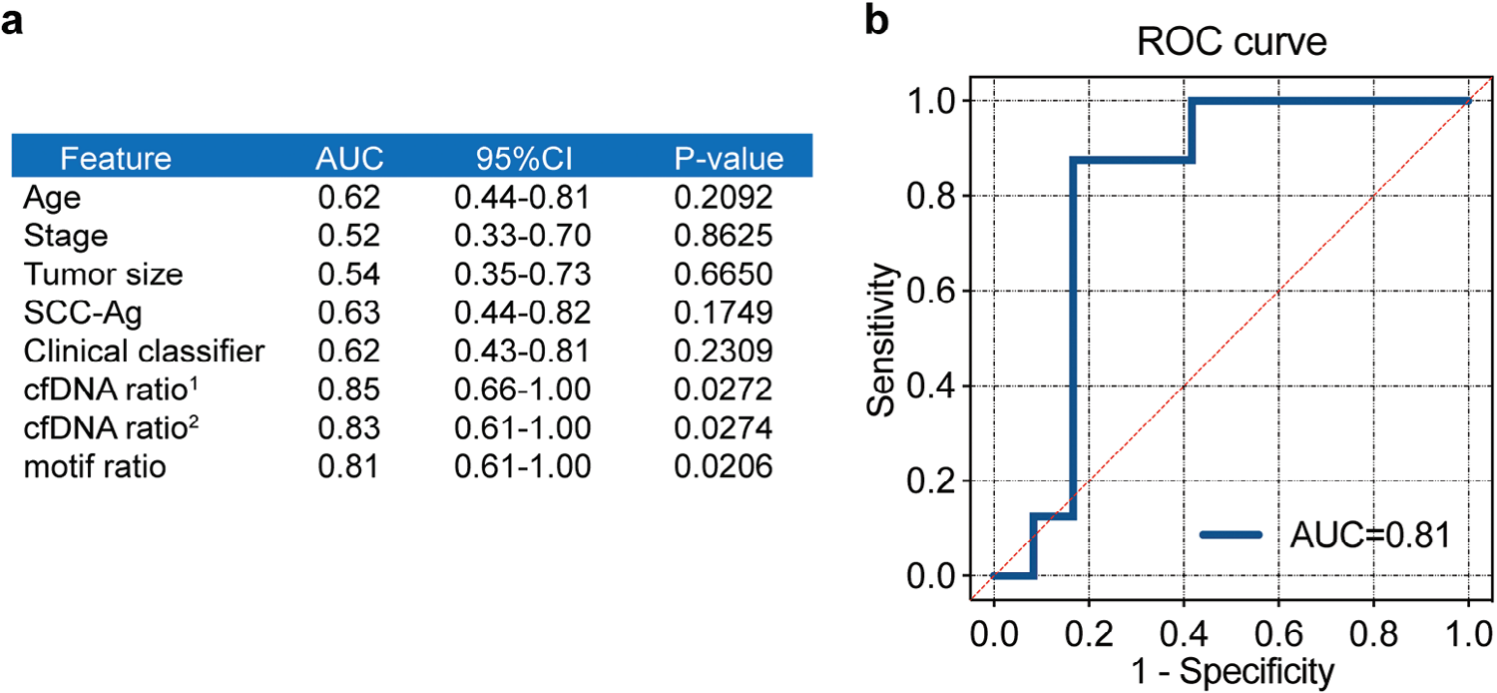
The use of clinical variables and cfDNA features in predicting non‐responders. a) Area under curves (AUCs) of clinical variables (age, tumor stage, tumor size, and SCC‐Ag, the clinical classifier) and cfDNA features (the cfDNA ratio and the motif ratio) in identifying non‐responders in cervical cancer patients. b) Receiver operator characteristic (ROC) curves of the motif ratio for discriminating the cervical cancer patients not responding to NACT by WGS.

To explore the value of clinical and cfDNA features in predicting NACT outcome, we used ROC curves analysis to evaluate the clinical validity of clinical variables (age, tumor stage, tumor size, and SCC‐Ag) and cfDNA parameters (the cfDNA ratio and the motif ratio) for discriminating the cervical cancer patients not responding to NACT. When predicting NACT response using baseline clinical variables in all non‐responders (*n* = 15) and responders (*n* = 24) across both cohorts, the AUCs for age, tumor stage, tumor size, and SCC‐Ag were notably lower, ranging from 0.52 to 0.63. Multiple logistic regression was used to build the predictive model of NACT response based on four clinical variables: age, stage, tumor size, and serum SCC‐Ag levels (pretreatment). The clinical classifier showed a poor discriminative capacity for the prediction of non‐responders from responders with an AUC of 0.62 (95% CI, 0.43–0.81) (**Figure** **6**a). In comparison, as shown in Figure 6a, the cfDNA ratio demonstrated good clinical performance in both cohort 1 and cohort 2, with an AUC of 0.85 and 0.83, respectively. The predictive discriminatory performance of the motif ratio was also good in the initial plasma of non‐responders (*n* = 8) and responders (*n* = 12) from cohort 2 (AUC = 0.81) (Figure 6b). Figure 4e validated the motif ratio's potential in distinguishing between responders and non‐responders in cohort 1.

### 2.7 Intracellular and Extracellular DNASE1L3 Activity in CC Patients

**Figure 7:**
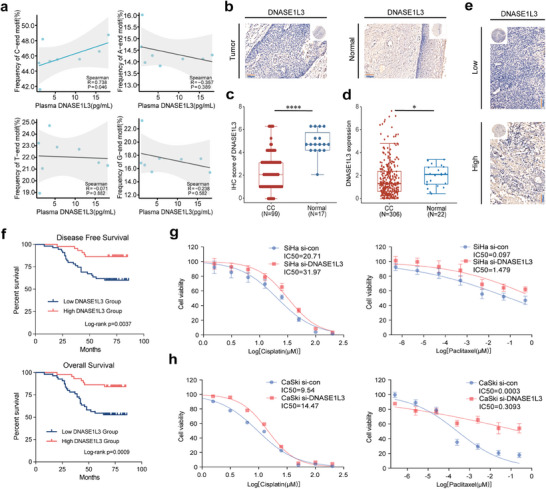
DNASE1L3 was a critical factor influencing tumorigenesis, progression, and the shaping of cfDNA characteristics. a) Correlation between plasma DNASE1L3 level and the frequencies of cfDNA molecules with different ending nucleotides in cervical cancer patients (*n* = 8). b) Representative immunohistochemical staining of DNASE1L3 in cervical cancer tissues and normal cervical tissues. c) DNASE1L3 expression levels in normal and tumor cervical tissue (99 cases of cervical cancer tissues and 17 cases of normal cervical tissues). The *p* values were determined by the Mann–Whitney U test: ns, not significant; *p < 0.05; **p < 0.01; ***p < 0.001; ****p < 0.0001. d) DNASE1L3 expression levels of tumor tissues from 306 cervical cancer patients from The Cancer Genome Atlas (TCGA) and normal cervical tissue from 22 subjects. The *p* values were determined by the Wilcoxon test: ns, not significant; *p < 0.05; **p < 0.01; ***p < 0.001; ****p < 0.0001. e) Representative immunohistochemical staining of cervical cancer tissues with high and low DNASE1L3 expression. f) Comparisons of DFS and OS in cervical cancer patients from the institutional dataset with low (*n* = 55) and high (*n* = 44) DNASE1L3 expression in tumor tissues. (*p* = 0.0037 and 0.0009, respectively by the log‐rank test). g) Viability of SiHa cells with (SiHa si‐DNASE1L3) and without (SiHa si‐con) siRNA directed against DNASE1L3 after treatment with cisplatin/paclitaxel in vitro. h) Viability of CaSki cells with (CaSki si‐DNASE1L3) and without (CaSki si‐con) siRNA directed against DNASE1L3 after treatment with cisplatin/paclitaxel in vitro.

It has been proved that DNASE1L3 plays a significant role in the degradation of plasma cfDNA and the generation of cfDNA with the C‐terminal end.^[^ ^25^ ^]^ Using ELISA, we measured the DNASE1L3 level in the initial plasma of eight cervical cancer patients (See Method). As presented in **Figure** **7**a, the proportion of cfDNA molecules with a C‐terminal end has a significant positive correlation with the DNASE1L3 level (Spearman r = 0.738, *p* = 0.046). There was no significant correlation between the frequencies of cfDNA with A/T/G fragments and DNASE1L3 levels in plasma. This observation validated the role of DNASE1L3 in shaping the cfDNA characteristic in humans. Considering that there is a significant downregulation of motifs starting with “C” in non‐responders (Figure 7a), we further explored the expression of DNASE1L3 in cervical cancer and its impact on chemotherapy sensitivity and tumor progression. A tissue microarray (TMA) was employed to assess the expression and prognostic significance of *DNASE1L3* in tumor (*n* = 99) and normal (*n* = 17) cervical tissues of cervical cancer patients (Institutional dataset, See Experimental Section). Figure 7b displays representative immunohistochemical staining of *DNASE1L3* expression in tumor and normal cervical tissue. According to Figure 7c, the tumor tissues exhibited significantly lower IHC scores of *DNASE1L3* than the normal tissues (median: 2.0 vs 4.5, *p*‐value < 0.0001), indicating a significantly reduced *DNASE1L3* expression level in tumor tissues. We further analyzed the transcriptome data of the tumor tissue of 306 cervical cancer patients from The Cancer Genome Atlas (TCGA) and normal cervical tissue from 22 subjects (3 from TCGA and 19 from Genotype‐Tissue Expression (GTEx)). The results showed a similarly significantly lower *DNASE1L3* expression in cervical cancer samples when compared to normal cervical samples (Figure 7d), which suggested that the dysregulation of *DNASE1L3* may play a role in the development and progression of cervical cancer.

Then patients were stratified into high and low *DNASE1L3* expression groups according to a cut‐off value of 3. Representative immunohistochemical staining of high *DNASE1L3* expression (IHC score ≥ 3) and low *DNASE1L3* expression (IHC score < 3) were shown in Figure 7e. Survival analysis on the institutional dataset revealed that the low expression of *DNASE1L3* in tumor tissue is correlated with a significantly decreased disease‐free survival (DFS) (Log‐rank *p* = 0.0037) and overall survival (OS) (Log‐rank *p* = 0.0009) of the patients, as depicted in Figure 7f. Additionally, we analyzed the prognosis of patients from the TCGA dataset, stratifying them into high (*n* = 73, top 25%) and low (*n* = 73, bottom 25%) *DNASE1L3* expression groups. Consistently, patients with low *DNASE1L3* expression showed decreased DFS (Log‐rank *p* = 0.015) and OS (Log‐rank *p* = 0.02) (Figure S5a,b, Supporting Information). To further investigate the functional significance of *DNASE1L3* expression in chemotherapy response, we performed a siRNA knockdown experiment on *DNASE1L3* of two cervical cancer cell lines. The results confirmed that low expression of *DNASE1L3* led to resistance to paclitaxel/cisplatin, as demonstrated by cell survival assays conducted on SiHa (Figure 7g) and CaSki cell lines (Figure 7h).

## 3 Discussion

Our research contributes significantly to understanding the impact patterns of two major cancer treatment methods, surgery and chemotherapy, on cfDNA levels, a facet not thoroughly explored in previous studies. We demonstrate that the tumor response to NACT is accurately reflected in timely changes in cfDNA levels, offering a promising avenue for quick monitoring in cervical cancer patients. The utilization of the cfDNA ratio provides a practical and cost‐effective means to obtain a snapshot of therapeutic outcomes. Notably, it enables the evaluation of chemotherapy sensitivity 6 weeks in advance, surpassing the capabilities of conventional clinical imaging and serum tumor markers. Furthermore, we have characterized the end motifs of initial plasma cfDNA fragments from both responders and non‐responders, identifying 23 representative differential motifs. Of particular significance is our discovery that the motif ratio encapsulates intrinsic characteristics of non‐responders, facilitating the distinction of chemotherapy sensitivity among patients. The application of motif ratio yields crucial insights into classifying responders and non‐responders, thereby enabling early identification of individuals unlikely to respond to NACT. This non‐invasive and cost‐effective approach holds immense potential in refining treatment strategies and improving patient outcomes in cervical cancer.

Recently, cfDNA emerged as a promising type of biomarker for early cancer detection, treatment response monitoring, and prediction of postoperative recurrence.^[^ ^11^ ^]^ However, its utility can be confounded by clinical treatments and associated complications, potentially obscuring disease‐related indicators. It's crucial to note that our study provides initial evidence of the variation pattern of cfDNA levels at different time points during treatment, thereby contextualizing our subsequent findings. We identified surgical trauma, chemotherapy, infection, and bone marrow depression as critical factors influencing cfDNA levels. Furthermore, we observed no significant alteration in cfDNA levels one day after surgery, but a notable increase from 2 to 7 days post‐surgery. These distinct patterns of cfDNA level variation at different time points during surgery may stem from the dynamics of cfDNA release. In contrast, chemotherapy induces a significant increase in cfDNA levels on day 1 after treatment, followed by a return to baseline before the next cycle. Importantly, we found that the cfDNA ratio effectively evaluates patient responses to NACT at an early stage. Total cfDNA levels and their fold change account for factors such as tumor dynamics and alterations in the tumor microenvironment. Previous studies support the notion that measuring cfDNA may offer a more efficient means of predicting treatment response,^[^ ^12^, ^13^, ^14^ ^]^ corroborating our findings and bolstering the robustness of our conclusions.

In recent years, cell‐free DNA fragmentomics have been proven to carry signals to its tissue‐of‐origin and show promising utility in cancer detection.^[^ ^40^ ^]^ However, their potential in predicting therapy response has remained unclear. In this study, we identified specific end motif patterns in non‐responders. These motifs were consistently observed in cfDNA sequenced using various protocols and sequencing strategies. Remarkably, these specific motifs enabled us to predict non‐responders in cfDNA from plasma samples before clinical treatment. Additionally, dysregulated biological processes and functions linked to chemotherapy resistance in non‐responders were uncovered through cfDNA TSS coverage analysis, providing further insights into the biological mechanisms underlying chemotherapy response. These findings underscore the potential of cfDNA fragmentomes as reliable non‐invasive biomarkers for predicting NACT response before treatment.

Based on the pivotal role of DNASE1L3 in cfDNA digestion and the generation of cfDNA with the C‐terminal end reported in the recent literature.^[^ ^18^, ^25^ ^]^ To further validate the intrinsic associations between the altered cfDNA characteristics (e.g., elevated cfDNA level, decreased “C”‐starting end sequence motifs) and chemotherapy sensitivity in cervical cancer, we conducted in vitro experiment and prognosis analysis. The positive correlation between DNASE1L3 activity and “C”‐starting end sequence motifs was confirmed in humans. Additionally, we revealed a significant association between the down‐regulated DNASE1L3 in cervical cancer and chemotherapy resistance and poor prognosis. These findings shed important light on the intrinsic associations between the altered cfDNA characteristics (e.g., elevated cfDNA level, decreased cfDNA size, reduced CCCA motif frequency) reported in cancer patients and the change of biological processes during tumorigenesis and progression.^[^ ^12^, ^13^, ^14^, ^18^, ^40^, ^41^ ^]^ Taken together, these findings highlight the potential mechanisms underlying our observations.

The major limitation of this study is the small sample size, further validation using larger cohorts from multiple centers is necessary to confirm these biomarkers clinical translation. However, it is worth noting that the results from both cohorts mutually corroborated our findings, and in vitro experiments further reinforced the robustness of our discoveries. Additionally, due to insufficient data on patient survival outcomes, we were limited in assessing the associations between the outcomes of the CC patients and the cfDNA characteristic at the early stage of the therapy. Therefore, further long‐term follow‐up and continuous investigation are required to address this issue.

In summary, our study lends support to the promising clinical utility of the cfDNA ratio and cfDNA fragmentomes, which encompass end motifs and TSS coverage, for predicting tumor response to NACT in cervical cancer and for monitoring treatment response. Future study protocols will hopefully validate the association between cfDNA characteristics and patient response

## 4 Experimental Section

### Patient Selection and Plasma Sample Collection

In this study, a total of 285 plasma samples were collected from 84 patients with histologically confirmed diagnoses of cervical cancer from the Department of Gynecologic Oncology of Tongji Hospital attached by the Tongji Medical College, Huazhong Science and Technology University (Wuhan, China). The sampling time points included both before and following any treatment administered during the patient's hospitalization, if available. (T1 to T14, see details in Figure 1 and Figure S1, Supporting Information). These patients were divided into two cohorts according to time sequences and employed different experimental methods and sequencing strategies to mutually validate the findings. For cohort 1, there were 45 patients with clinically and histologically confirmed CC recruited between 2019 and 2020, including 22 patients (Stage IB3–III) receiving platinum‐based NACT. Among them, 19 patients who received postoperative adjuvant chemotherapy were derived from a clinical trial at Tongji Hospital of Wuhan in China (ClinicalTrials.gov numbers, NCT01755897). In total, 179 blood samples were collected from the initial time point (i.e., before treatment) to the end of the treatment from these subjects (Figure S1A, Supporting Information). Cohort 2 consists of 39 patients with CC, from whom 106 plasma samples were collected from 2021 to 2022. Among the patients in cohort 2, 24 individuals with Stage IB3–III CC received NACT (Figure S1B, Supporting Information). Peripheral blood samples were collected using EDTA tubes and were centrifuged within 4 h of collection at 1600 g for 10 min at 4 °C followed by centrifuging again at 16 000 g for 15 min at 4 °C. All plasma samples were collected and stored at −80 °C. This study was approved by the Ethics Committee of Tongji Hospital in Wuhan (TJ‐IRB20200704) and The Institutional Review Board of BGl (BGI‐IRB 22139). All patients gave written informed consent. The study was conducted following the principles outlined in the Declaration of Helsinki.

### cfDNA Quantification in Plasma and Next‐Generation Sequencing

For cohort 1, cfDNA extraction and quantification were performed in 179 plasma samples of cohort 1 by using a standard commercial kit following the manufacturer's instructions (The QIAamp ccfDNA/RNA Kit, Qiagen, Cat. No. 55184). The cfDNA concentration was measured by Qubit dsDNA HS Assay Kit (Q32854, Invitrogen). Among them, 70 plasma samples meeting the sequencing requirements (total cfDNA < 25 ng) from 18 patients (P01–P18) were additionally processed for library preparation and whole‐exome sequencing (WES) to evaluate the molecular characteristics of cell‐free DNA. The libraries were constructed following the standard protocols provided by Illumina. Exome capture was performed using SureSelectXT Human All Exon V6 (Agilent) technology. The sequencing was conducted using an Illumina NovaSeq6000 platform in a 150 bp × 2 paired‐end format. For cohort 2, it used the different methods of quantification and sequencing of cfDNA to examine the consistency of the results from cohort 1 and analyze the fragmentation patterns of cell‐free DNA across the genome. The MagPure Circulating DNA Kit (Cat. No. MD5432‐02) and the MGIEasy Cell‐free DNA Library Prep Set (excluding the MGIEasy Rapid Circularization Module, Cat. No. 1000005258) on the MGISP‐960 platform were utilized for quantification and library preparation of cfDNA in cohort 2. Subsequently, all plasma samples successfully underwent whole genome sequencing (WGS) of cfDNA using the DNBSEQ platform (MGI) in a 100 bp × 2 paired‐end format. The cfDNA ratio was calculated by dividing the cfDNA level at day 1 after the first cycle of NACT (T2) by the cfDNA level prior to treatment (T1).

### Sequencing Data Processing and Alignment

For quality control, Fastp (v0.20.1)^[^ ^42^ ^]^ was utilized to trim the sequencing adaptor and eliminate low‐quality reads from the raw sequencing data. Subsequently, the cleaned reads were aligned to the human reference genome (hg38) using minimap2 (v2.11‐r797‐v07).^[^ ^43^ ^]^ PCR duplications were identified using bamsormadup (v2.0.79, https://github.com/gt1/biobambam2) and subsequently removed. Reads with low mapping quality (< 30), multiple alignments, and those with more than five mismatches were filtered out. Only paired‐end reads with proper mapping orientations and an insert size below 600 bp were retained for downstream analysis.

### Coverage Analysis in the Promoter Region

The genomic locations of the transcriptional start site (TSS) were downloaded from UCSC (https://hgdownload.soe.ucsc.edu/goldenPath/hg38/bigZips/p13/). The core region was defined as a 500 bp genome region surrounding TSS, and the flanking region as the regions spanning 250 to 500 bp upstream and 250 to 500 bp downstream in relation to TSS.^[^ ^44^ ^]^ TSS scores were further calculated as the ratio between the mean depth of the core region and the flanking region. The TSS scores were utilized to evaluate the TSS coverage profile of each gene in the plasma DNA. A high TSS score signifies a low expression level of the gene, whereas a low TSS score indicates a high gene expression level.

### Measurement of Tumor Size and Classification of Patients with NACT as Responder or Non‐Responder

The patient's chemotherapy regimen was paclitaxel (165–175 mg m^−2^) and cisplatin (75–80 mg m^−2^) once every 3 weeks. The tumor size was evaluated by vaginal ultrasound before each cycle of treatment and verified with Magnetic resonance imaging (MRI). The surgical pathology results after NACT were also examined to confirm the NACT response. The percentage of tumor shrinkage was measured between the initial tumor size and the size of the tumor after two cycles of NACT and evaluated using the RECIST criteria. This criterion categorizes the response as follows: complete resolution of measurable tumor (CR, complete response), a decrease of ≥ 30% in the largest tumor diameter (PR, partial response), the emergence of new lesions, or an increase of ≥ 20% in tumor size (PD, progressive disease), and any cases that do not meet either the PR or PD criteria (SD, stable disease). Patients who received NACT were then classified as responders (CR+PR) or non‐responders (SD+PD). Furthermore, a pathological complete response (pCR) was defined as the absence of residual invasive tumors within the lesions.

### Quantification of the Level of DNASE1L3 in Plasma

DNASE1L3 levels in plasma were measured using an ELISA kit according to the manufacturer's protocol (Cusabio, Wuhan, China).

### DNASE1L3 Knockout and Drug Sensitivity Detection

A siRNA (5 nm) against *DANSE1L3* (RiboBio) was transfected into cervical cancer cell lines (SiHa and CaSki) in 6‐well plates using Lipofectamine 3000 (Invitrogen) according to the manufacturer's instructions. Gene silencing was measured by Western blot analysis 72 h after transfection. For drug sensitivity measurements, a Cell Counting Kit‐8 (Dojindo) was used to detect the viability of cells. Transfected cells were replicated more than three times in 96‐well plates at a density of 5 × 10^3^ cells per well. Then twofold increasing concentrations of cisplatin (0 to 500 µm) or tenfold paclitaxel (0 to 0.5 µm) were applied to the cells for 48 h. The cell viability was quantified with the absorbance at 450 nm in a SpectraMax Microplate reader (Molecular Devices). The data were analyzed using GraphPad Prism 9 software.

### DNASE1L3 Expression and Prognosis Analysis

A tumor microarray slide (HUteS136Su01) contained 99 specimens of cervical cancer tissues and 17 specimens of normal cervical tissues collected by Shanghai Outdo Biotech Company (the ethics approval number: SHYJS‐CP‐1801014). Immunohistochemistry for *DNASE1L3* was performed to determine the expression. The slide was first deparaffinized and rehydrated. The antigens were then repaired with EDTA (pH 9.0) and the endogenous peroxidase was blocked with 3% hydrogen peroxide. The slide was blocked with 3% BSA (G5001‐100 g, Servicebio) at room temperature for 1 h and then incubated with primary antibody (DNASE1L3, ab152118, Abcam, 1:400 dilution) at 4 °C overnight. After primary antibody exposure, the slide was sequentially incubated with biotinylated goat anti‐rabbit IgG (G1215‐3, Servicebio, 1:100 dilution) for 1 h at room temperature and then streptavidin‐peroxidase conjugate for 30 min at room temperature. Finally, the slide was subjected to DAB staining, nuclear restaining, and dehydration. After the experiment, the staining intensity and staining range of each site on the slide were evaluated by professional pathologists to calculate the protein expression level of *DNASE1L3*. Based on the intensity of immunostaining, the scores were: 0, no staining; 1, weak staining; 2, moderate staining; 3, strong staining. The staining area score was defined as 0 (no staining area), 1 (lower 30%), 2 (30–70%), and 3 (higher 70%). *DNASE1L3* protein expression of each specimen was quantified as a result of the staining intensity score multiplied by the staining area score. Survival analysis of *DNASE1L3* in cervical cancer was performed using the Kaplan Meier method by GraphPad Prism 9 software. The RNA‐seq data from 306 cervical tumor tissue and 22 normal cervical tissue were obtained from TCGA (https://www.cancer.gov/ccg/research/genome‐sequencing/tcga) and GTEx project (https://gtexportal.org/home/) and compared *DNASE1L3* expression between the tumor and normal tissues. Survival analysis of *DNASE1L3* for cervical cancer patients from TCGA was performed with the GEPIA2 (http://gepia2.cancer‐pku.cn/) analysis tool.^[^ ^45^ ^]^

### Predictive Performance of Various Features by ROC Curve Analysis

Receiver operating characteristic curve analysis, implemented in the GraphPad Prism 9 software, was employed to evaluate the predictive performance of NACT response based on cfDNA features (cfDNA ratio, motif ratio, and TSS ratio) and clinical variables (age, clinical stage, initial tumor size, and SCC‐Ag level). In detail, age was divided into two categories (≤ 50/> 50). The clinical stage was divided into two categories (< IIB/≥ IIB). Initial tumor size was divided into two categories (< 4 cm/≥ 4 cm).

### Statistical Analysis

The Mann–Whitney U‐test, Wilcoxon test, and the Kruskal–Wall test were applied for the determination of significant differences among different groups for continuous variables. The difference in categorical variables between groups was examined by the Chi‐square test or Fisher's exact test. Statistical significance was determined through a threshold of a *p*‐value below 0.05. The symbols of “*”, “**”, “***”, and “****” in the figures indicate the *p* values less than 0.05, 0.01, 0.001, and 0.0001, respectively. All statistical analysis was conducted by the Scipy (version 1.7.1) module of python3.

## Conflict of Interest

The authors declare no conflict of interest.

## Author Contributions

T.P., H.Z., L.L., and C.C. contributed equally to this work. P.W., X.J., W.D., and J.Y. conceived and designed the study and managed the project. T.P., C.C., Y.W., Y.Z., X.L., and M.X. collected and processed samples. T.P., H.Z., L.L., Y.Z., Y.W., and C.C. designed, performed, and analyzed in vitro experiments and wrote the original draft of the manuscript. T.P., H.Z., and L.L. accessed and verified the original data. H.Z. and L.L. were responsible for directing the statistical analysis. T.P., H.Z., L.L., C.C., M.X., X.L., P.W., S.L., T.C., B.L., Y.X., and P.W. supervised the project. The manuscript was drafted and revised by T.P., H.Z., L.L., C.C., and P.W. and was reviewed by all authors.

## Supporting information



Supporting Information

## Data Availability

The data that support the findings of this study are available from the corresponding author upon reasonable request.
