# Supplementary material for: Plasma Cell‐Free DNA Concentration and Fragmentomes Predict Neoadjuvant Chemotherapy Response in Cervical Cancer Patients
Source: Adv Sci (Weinh). 2024 Sep 25;11(43):2309422. doi: 10.1002/advs.202309422 (PMC11578340; doi:10.1002/advs.202309422)
Supplement: Supplementary file 1 — Supporting Information [file ADVS-11-2309422-s001.docx]

Supporting Information

Plasma cell-free DNA concentration and fragmentomes predict neoadjuvant chemotherapy response in cervical cancer patients

Ting Peng, Haiqiang Zhang, Lingguo Li, Canhui Cao, Miaochun Xu, Xiaojie Liu, Shitong Lin, Ping Wu, Tian Chu, Binghan Liu, Yashi Xu, Yan Zhang, Yeqin Wang, Jinjin Yu*, Wencheng Ding*, Xin Jin*, and Peng Wu*


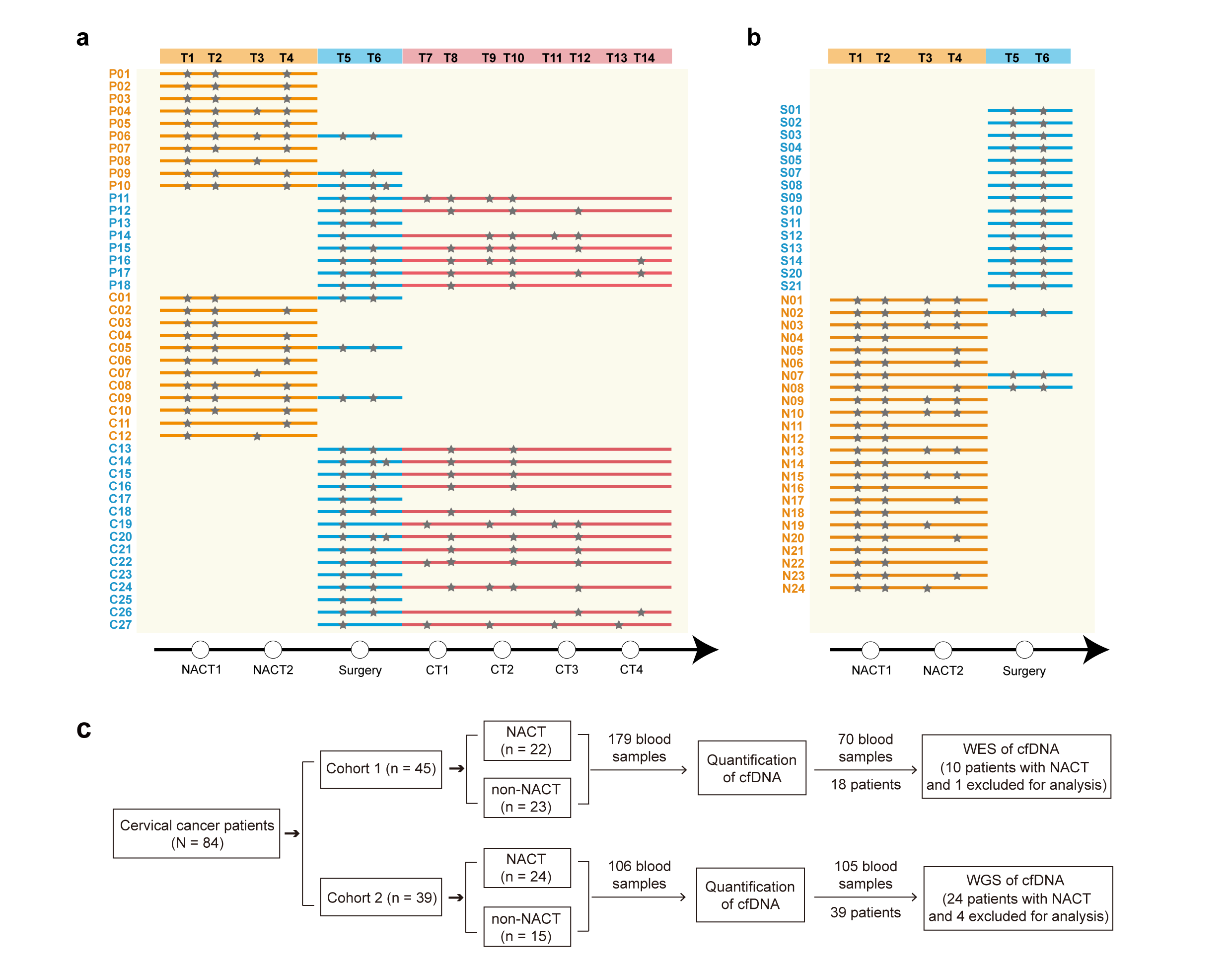


**Figure S1.** The time points of sample collection. a) Cohort 1. b) Cohort 2. The orange, blue, and red timelines indicate the sample collected during NACT, surgery, and post-surgery chemotherapy phase, respectively. Orange numbers indicate patients with NACT and blue numbers indicate patients without NACT. c) The breakdown of the study population.


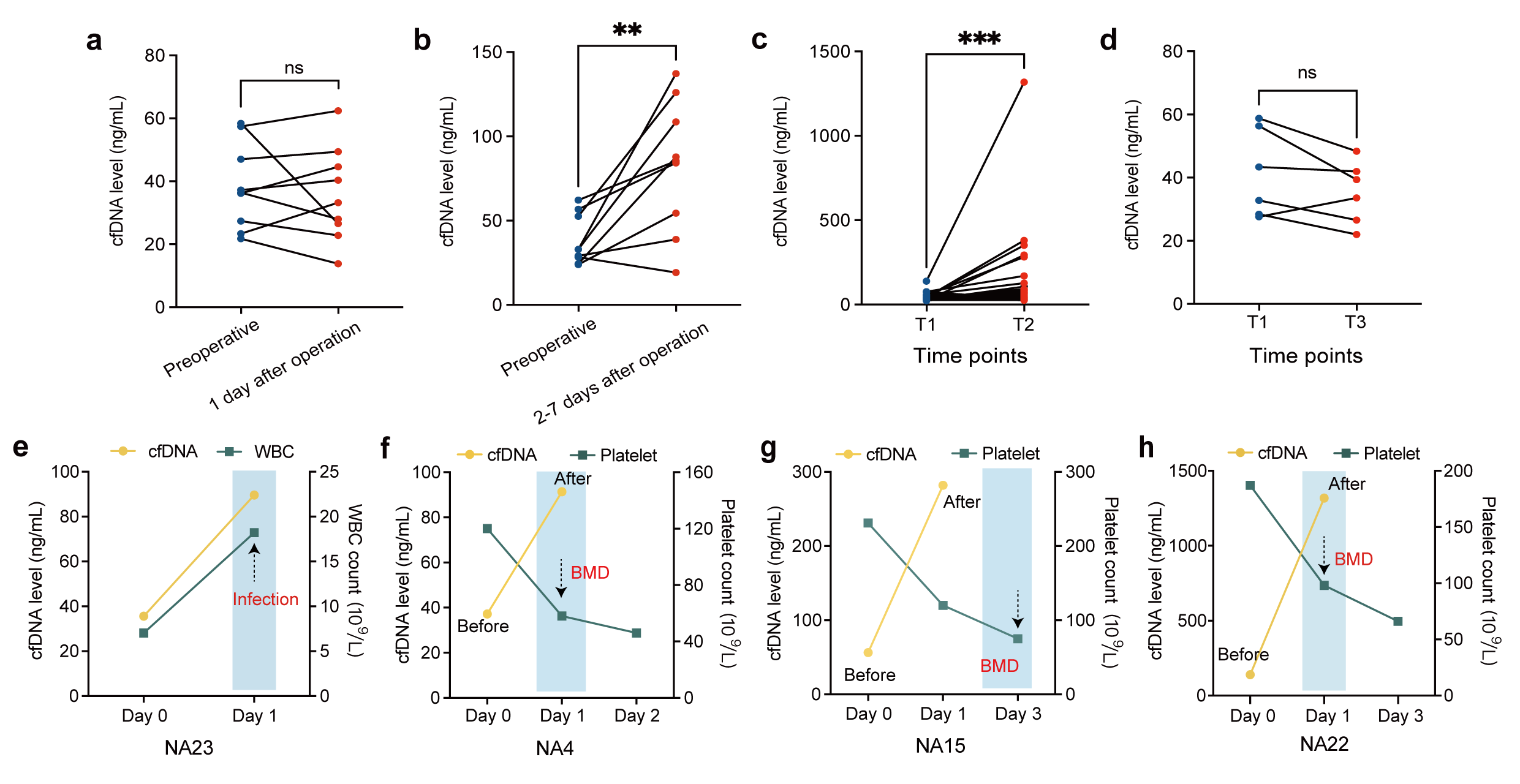


**Figure S2.** The cfDNA level during surgery and chemotherapy in cohort 2. a) Comparison of cfDNA levels one day after surgery to preoperative levels (*n* = 9). b) Comparison of cfDNA levels 2 to 7 days after surgery to preoperative levels (*n* = 9). c) Comparison of cfDNA levels between day 1 after the first cycle of NACT and pre-NACT (*n* = 24). d) Comparison of cfDNA levels between day 1 after the second cycle of NACT and pre-NACT (*n* = 5). e) Patient NA23: cfDNA levels during chemotherapy treatments, exhibiting a significant rise in the context of infection. f–h) Three patients (NA04, NA15, NA22): cfDNA levels during chemotherapy treatments, showing a significant elevation in the presence of bone marrow depression (BMD). The *p* values were determined by the Wilcoxon test: ns, not significant; **p* < 0.05; ***p* < 0.01; ****p* < 0.001; *****p* < 0.0001.


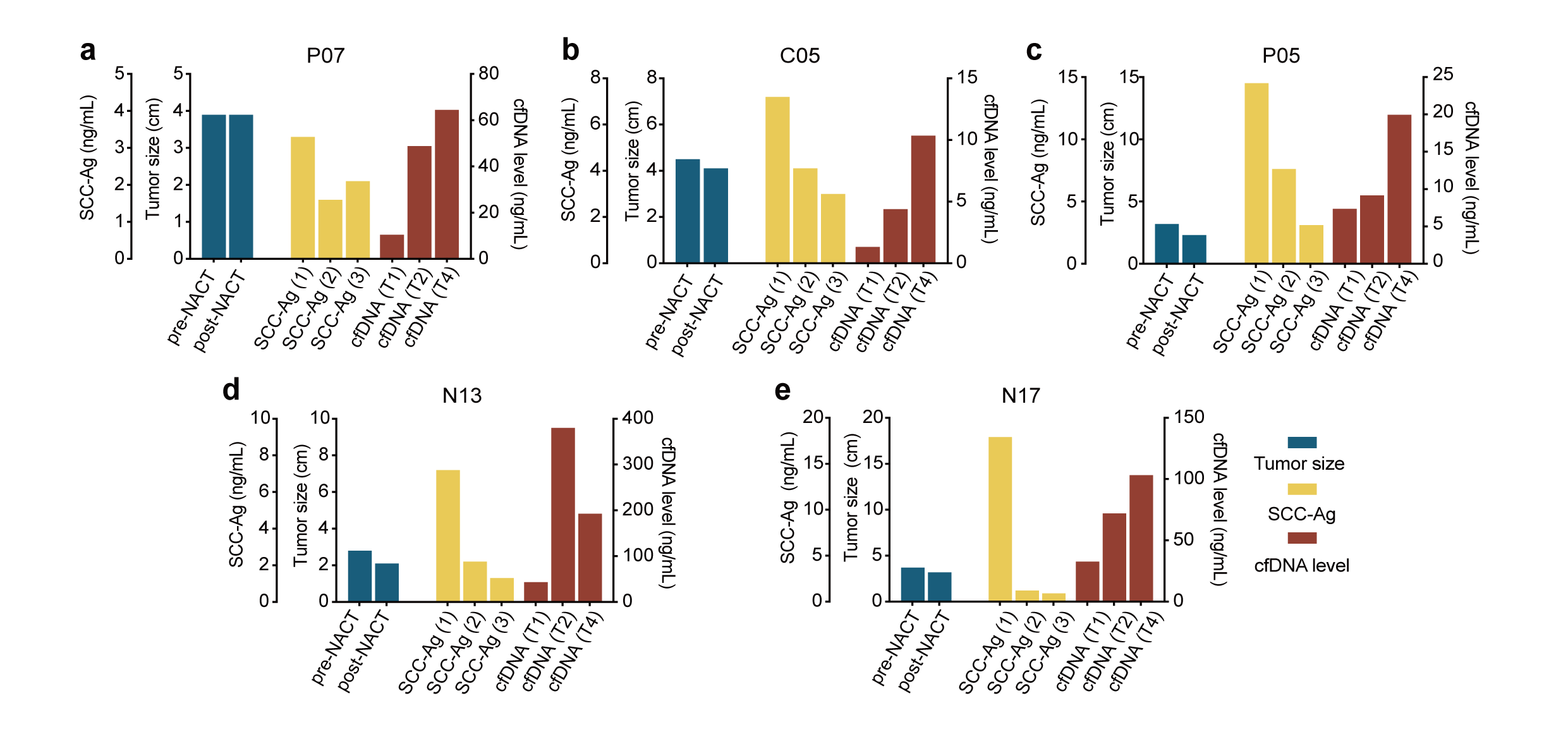


**Figure S3.** Longitudinal monitoring of cfDNA level in non-responders. a–c) Three typical non-responder patients (P07, C05, and P05) of cohort 1 with an abnormal decrease in SCC-Ag level after treatment and a continuous increase in cfDNA level at day 1 after each chemotherapy. d–e) Two non-responder patients (N13, N17) of cohort 2 with an abnormal decrease in SCC-Ag level exhibited elevated cfDNA levels.


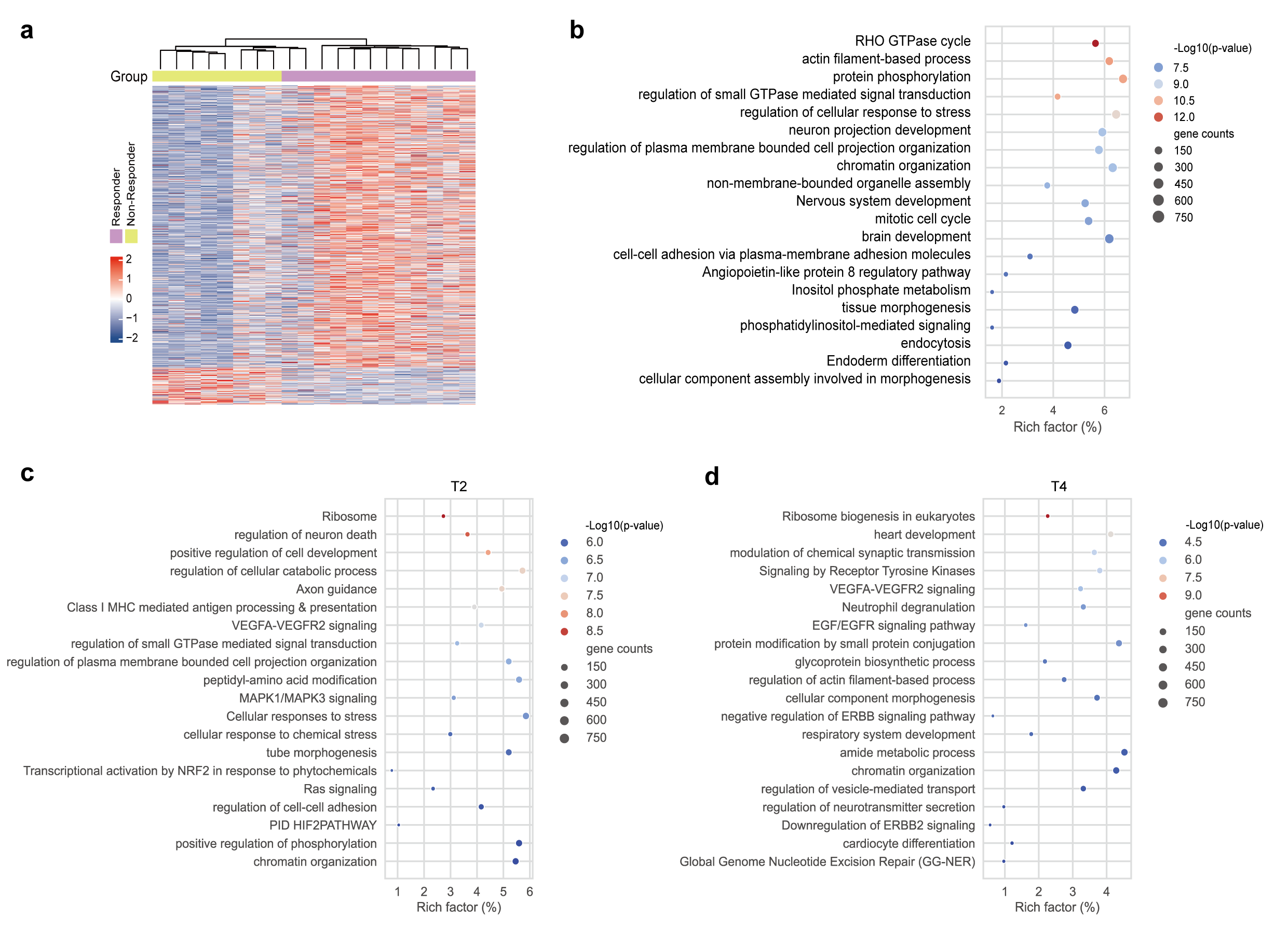


**Figure S4.** a) The unsupervised clustering of the differentially expressed genes among patients with and without NACT response based on TSS scores. b) The top 20 enriched pathways of the dysregulated genes reported by Xun *et al*. are associated with chemotherapy resistance in patients undergoing NACT. c) Enriched pathways of inferred differential genes in the T2 time point. D) Enriched pathways of inferred differential genes in the T4 time point.


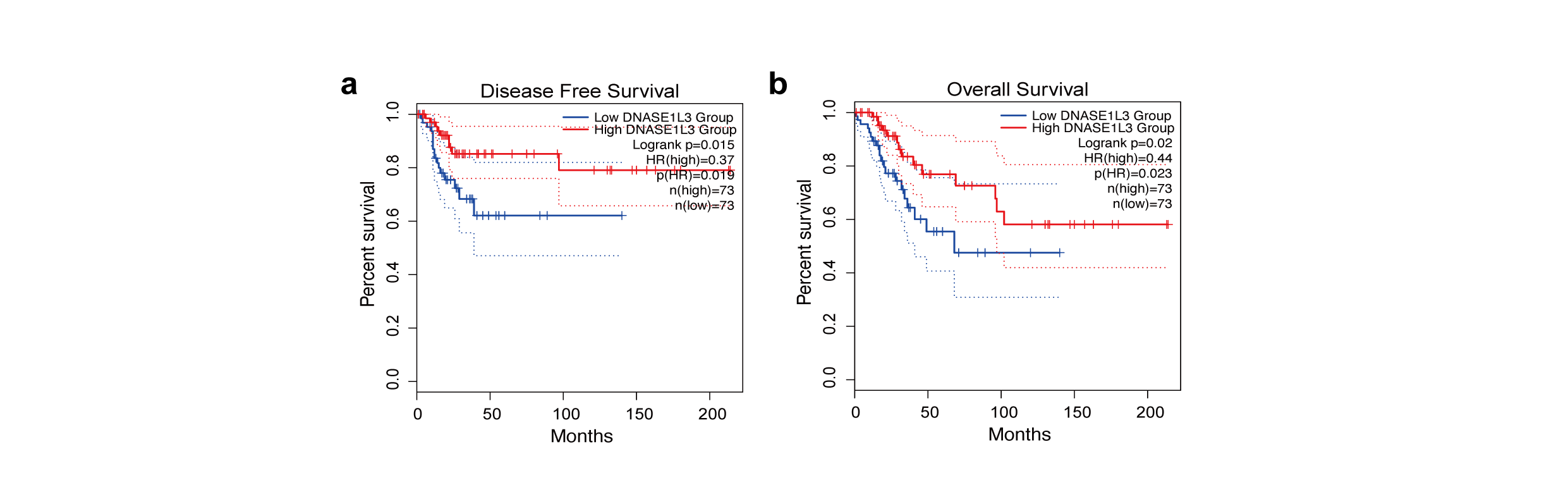


**Figure S5.** a) The disease-free survival analysis of the expression of DNASE1L3 in cervical cancer patients (*n* = 146) from the TCGA dataset (log-rank test, *p* = 0.015). b) The overall survival analysis of the expression of DNASE1L3 in cervical cancer patients (*n* = 146) from the TCGA dataset (log-rank test, *p* = 0.02).
